# Radiological Characteristics of Patients With Anti-MDA5–Antibody-Positive Dermatomyositis in ^18^F-FDG PET/CT: A Pilot Study

**DOI:** 10.3389/fmed.2021.779272

**Published:** 2021-11-22

**Authors:** Heng Cao, Junyu Liang, Danyi Xu, Yinuo Liu, Yinan Yao, Yiduo Sun, Ye He, Jin Lin

**Affiliations:** ^1^Department of Rheumatology, The First Affiliated Hospital, Zhejiang University School of Medicine, Hangzhou, China; ^2^PET Center, The First Affiliated Hospital, Zhejiang University School of Medicine, Hangzhou, China; ^3^Department of Respiratory and Critical Care Medicine, The First Affiliated Hospital, Zhejiang University School of Medicine, Hangzhou, China

**Keywords:** dermatomyositis, melanoma differentiation associated protein 5 (MDA5), ^18^F-fluorodeoxyglucose positron emission tomography/computed tomography (^18^F-FDG PET/CT), interstitial lung disease (ILD), standardized uptake value (SUV)

## Abstract

**Objective:** To elucidate the ^18^F-fluorodeoxyglucose (FDG) PET/CT characteristics and its prognostic value in the patients with anti-melanoma differentiation associated protein 5 antibody positive (anti-MDA5+) dermatomyositis (DM).

**Methods:** This retrospective cross-sectional study included 26 patients with anti-MDA5+ DM and 43 patients with anti-MDA5 negative (anti-MDA5–) idiopathic inflammatory myopathy (IIM) who were examined by ^18^F-FDG PET/CT from January 1, 2017 to December 31, 2020. The maximum standardized uptake value (SUVmax) of multiple organs and other clinical characteristics of the patients were measured and analyzed.

**Results:** Compared with the anti-MDA5– group, the patients in the anti-MDA5+ group showed higher bilateral lung SUVmax (*p* = 0.029), higher SUVmax of spleen (*p* = 0.011), and bone marrow (*p* = 0.048). Significant correlations between the spleen SUVmax and serum ferritin levels (*r* = 0.398, *p* < 0.001), erythrocyte sedimentation rate (ESR) (*r* = 0.274, *p* = 0.023), platelet count (*r* = −0.265, *p*= 0.028), myositis disease activity assessment score (*r* = 0.332, *p* = 0.005), bone marrow SUVmax (*r* = 0.564, *p* < 0.001), and bilateral lung SUVmax (*r* = 0.393, *p* < 0.001) were observed.

**Conclusion:** ^18^F-FDG PET/CT was found valuable in quantifying the pulmonary focal inflammation and potentially unveil the distinctive characteristics and pathophysiological mechanisms in the patients with anti-MDA5+ DM.

## Key Messages

1. ^18^F-FDG PET/CT was found valuable in quantifying the pulmonary focal inflammation in the patients with anti-MDA5+ DM.

2. High FDG uptake in spleen was associated with myositis disease activity and pulmonary inflammation.

## Introduction

Idiopathic inflammatory myopathies (IIM) are a heterogeneous group of autoimmune diseases, which display several clinical manifestations, such as muscle, skin, or articular involvement, interstitial lung disease (ILD), and sometimes associated with malignancy (1). The myositis-specific autoantibodies contribute to delineate the homogenous subgroups of IIM (2, 3). Dermatomyositis (DM) associated with anti-melanoma differentiation associated protein 5 antibody (anti-MDA5+) is typically defined by the presence of characteristic skin lesions (Gottron's papules, heliotrope rash) and ILD, whereas the clinical signs of myositis are usually mild or absent (4). Rapidly progressive ILD (RP-ILD), which is a serious and life-threatening condition, leads to high mortality in anti-MDA5+ DM (5–7).

The studies on the predictive risk factors for RP-ILD in the patients with anti-MDA5+ DM revealed that biomarker, such as ferritin, Krebs von den Lungen-6 (KL-6), MDA5 antibody titers, and baseline forced vital capacity (FVC) were considered to be important in evaluating the disease activity and prognosis (8, 9). The image techniques were widely used in the evaluation of anti-MDA5+ DM. High resolution computed tomography (HRCT) was often performed to verify the pathological classification of ILD in the patients with anti-MDA5+ DM patients (5, 10).

^18^F-fluorodeoxyglucose (FDG) PET/CT had proven to be a useful, hybrid technique (combining nuclear and CT imaging) for detecting the morphologic and functional changes in a variety of diseases. PET/CT was usually performed to screen malignancy in the patients with IIM (11). Moreover, abnormal increases in ^18^F-FDG uptake on the PET/CT images were observed in proximal muscles, which are correlated to inflammatory lesions in the patients with IIM (12, 13). A good correlation was also found between the maximal ^18^F-FDG standardized uptake value (SUVmax) and the results of muscle biopsy in the patients with myositis patients (14). Li et al. (15) reported that ^18^F-FDG uptake on the PET/CT images is observed not only in the proximal muscles but also in interstitial lung disease. In this study, high sensitivity and specificity for detecting rapidly progressive interstitial lung disease were reported when the ^18^F-FDG SUVmax was higher than 2.5 on PET/CT (15). Our previous study reported that higher ^18^F-FDG uptake of the interstitial lesions of the patients with IIM on PET/CT images is significantly associated with RP-ILD and poor outcome (16).

However, the PET/CT characteristics remain unclear in the patients with anti-MDA5 positive DM. Thus, this retrospective study was conducted to elucidate the PET/CT characteristics and their effect on the clinical course and its prognostic value in anti-MDA5+ DM. In addition, we compared the patients with anti-MDA5+ with a group of patients with IIM without anti-MDA5 antibody (anti-MDA5–) to confirm the specificity of the PET/CT characteristics.

## Methods

### Patients Population

This retrospective cross-sectional study was conducted in the First Affiliated Hospital, Zhejiang University School of Medicine (FAHZJU) in China. In-hospital databases were searched for ^18^F-FDG PET/CT scans of the patients with IIM that were performed between January 1, 2017 and December 31, 2020. The inclusion criteria for this study were: (1) age over 18 years old. (2) The diagnosis of DM, polymyositis (PM), or amyopathic dermatomyositis (ADM) was based on the 2017 the American College of Rheumatology (ACR) and the European League Against Rheumatism (EULAR) classification criteria (17). (3) A ^18^F-FDG PET/CT scan was performed during hospitalization. The exclusion criteria were: (1) newly identified or unsolved malignancies; (2) clarified overlap syndrome with other connective tissue diseases; (3) myopathy related to thyroid dysfunction, strenuous exercise, inherited metabolic disorders, and drug-induced myositis; (4) loss of follow-up for any cause within 3 months after hospitalization. A written informed consent was acquired from all the included patients and the study was approved by the Institutional Review Board of FAHZJU (No. IIT202100194).

### Clinical and Laboratory Data

All the clinical and biological data were collected at the time of the inclusion. Clinical information was independently collected by two rheumatologists. Data collected were demographics, course of disease, clinical manifestations, complications, laboratory results, pulmonary function, myositis disease activity assessment (MYOACT) (18), and autoantibody status. The myositis-specific autoantibodies (MSAs, anti-MDA5, anti-Jo-1, anti-OJ, anti-PL-7, anti-PL-12, anti-EJ, anti-TIF1γ, anti-Mi-2α, anti-Mi-2β, anti-NXP2, anti-SRP, and anti-SAE1) and myositis-associated autoantibodies (MAAs, anti-Ro-52, anti-PM-Scl75, anti-PM-Scl100, and anti-Ku) were identified using the immunodot assays (Euroimmun, Lübeck, Germany) according to the instructions of the manufacturer. All the included patients received immune-suppressive medications. The immunosuppressive regimens used during hospitalization were categorized into five groups: (1) steroid monotherapy; (2) steroid + disease-modifying antirheumatic drugs (DMARDs); (3) steroid + intravenous immunoglobulin (IVIG); (4) steroid + DMARDs +IVIG; (5) steroid + Janus kinase (JAK) inhibitors. JAK inhibitors mainly referred to tofacitinib and baricitinib. The DMARDs included the usage of cyclophosphamide, mycophenolate mofetil, cyclosporine, tacrolimus, azathioprine, methotrexate, thalidomide, and hydroxychloroquine.

### ILD and HRCT Imaging Assessments

In each patient with IIM, the HRCT scan was performed within 1 month before or after the ^18^F-FDG PET/CT scans. Thin-section CT images were obtained in the supine position during breath-holding and deep inspiration. The chest HRCT images were re-assessed by an experienced radiologist and a respiratory specialist who were blinded to the clinical information of the patients. The radiological ILD patterns were defined as per the American Thoracic Society/European Respiratory Society criteria, which was a multidisciplinary decision made by the rheumatologists, respirologists, and radiologists (19). RP-ILD was defined as follows in this study: previous or concurrent diagnosis of ILD, presenting with progressive dyspnea or hypoxemia, and a worsening of interstitial change on chest HRCT radiograph within 1 month after the initial visit or onset of respiratory symptoms (20, 21).

### FDG-PET/CT Imaging

^18^F-FDG PET/CT had to be performed within 2 weeks of diagnosis. The PET/CT acquisitions were carried out according to the European Association of Nuclear Medicine procedure guidelines (22). The patients were instructed to avoid exercise for at least 24 h and fast for at least 6 h prior to PET/CT detection. The mean serum glucose level was 5.5 mmol/L (range 5.2–6.1 mmol/L) and confirmed before the injection of 4.0 MBq/kg ^18^F-FDG. Then, 1 h after the intravenous injection, whole-body FDG-PET images were obtained using a dedicated PET scanner (Biograph, Sensation 16, Siemens systems, Germany). Low-dose CT was performed with 120 kV and 30 mAs prior to emission scanning. ^18^F-FDG uptake was calculated by the following formula: SUV (g/ml) = regional radioactivity concentration (Bq/ml)/[injected dose (Bq)/body weight (g)].

Visual examinations for the detection of increased ^18^F-FDG uptake were retrospectively performed by a single trained radiologist who was blinded to the diagnosis, complications, and outcome of all the patients involved. FDG uptake was calculated by using SUVmax of a circular region of interest (ROI) with a fixed diameter of 20 mm, containing lung, liver, spleen, bone marrow, heart, digestive system, cerebellum, and muscle. Bilaterally proximal muscle observed by PET/CT contained deltoideus, biceps brachii, trapezius, iliopsoas, gluteus maximus, gluteus medius, and quadriceps.

### Statistical Analysis

All the statistical analyses were performed using SPSS 22.0 (Chicago, IL, USA), R 3.6.1 and Graphpad Prism 8.0 (San Diego, CA, USA). The variables were expressed as mean ± SD or median (quartiles). The categorical variables were expressed as frequencies and percentages. The comparisons were performed by using the *t*-test or Mann–Whitney *U*-test for the quantitative variables. Unordered categorical variables were compared using the chi-square test or Fisher's exact. The survival curves were drawn by using the Kaplan–Meier method and compared by using the log-rank tests. The correlation between the two continuous variables was assessed utilizing Pearson's linear analysis. All the statistical tests were performed two-sided, and a *P*-value < 0.05 was considered statistically significant.

## Results

### Patient Characteristics

**Figure 1:**
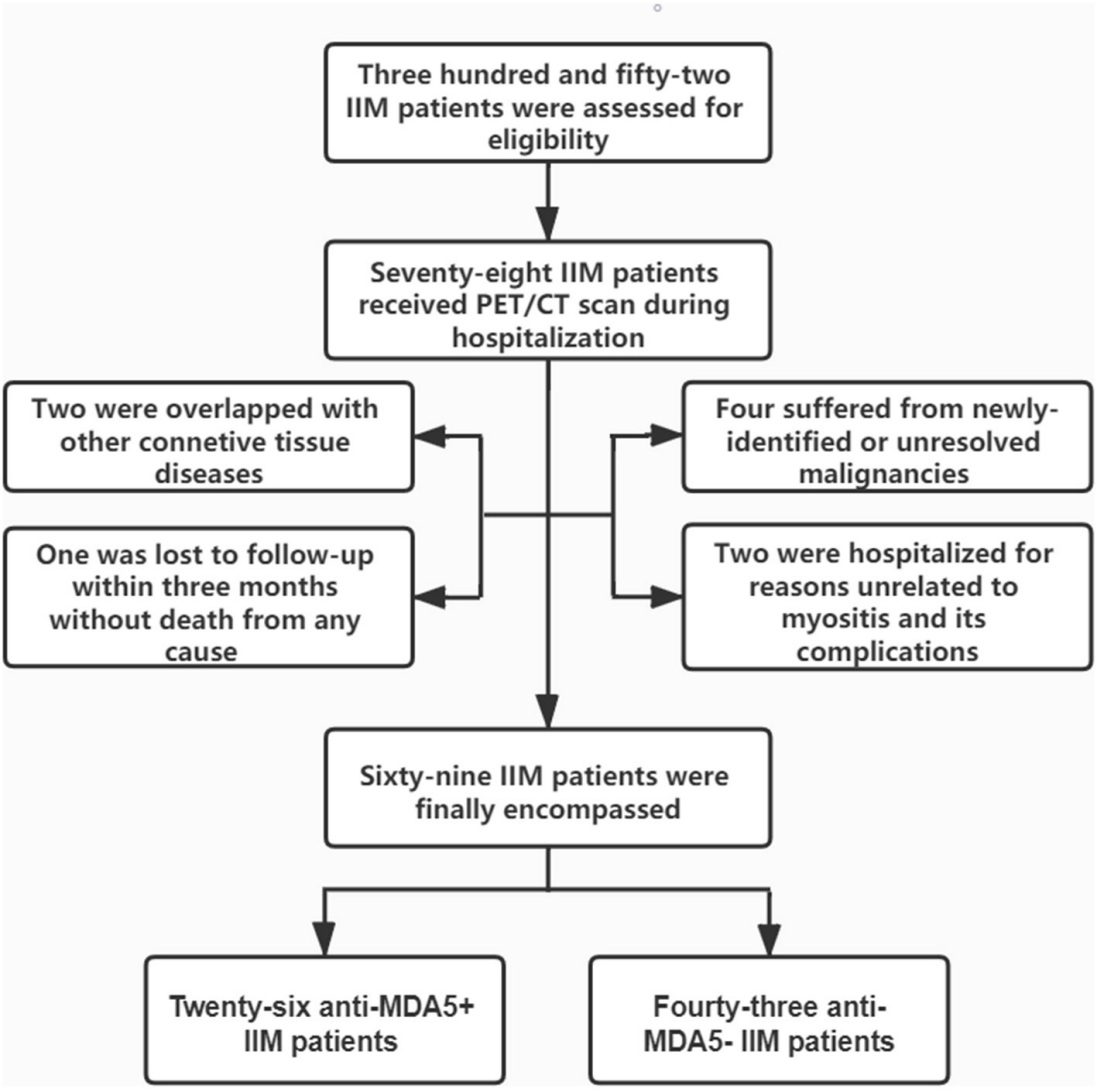
Enrollment and groupings of this study. IIM, idiopathic inflammatory myopathies; PET/CT, positron emission tomography/computed tomography; MDA5, melanoma differentiation associated protein 5.

A total of 69 patients who satisfied the inclusion/exclusion criteria were included in the study (Figure 1). As characterized in Table 1, 26 patients with anti-MDA5+ DM were incorporated into this study, encompassing 17 with DM and 9 with ADM. Eighteen of them (69.2%) were women and the mean age was 55.92 ± 7.68 years old. The medium follow-up time was 11.9 (4.00, 23.80) months. The anti-MDA5+ and the anti-MDA5– group were similar with respect to age, gender, course of disease, and duration of diagnosis delay. Among the patients with anti-MDA5+ DM, 53.8% had fever, 38.5% had splenomegaly, and 15.4% had gastrointestinal hemorrhage. We found no difference in the prevalence of co-existing infectious complications between the anti-MDA5+ and the anti-MDA5– group. However, the prevalence of preceding carcinoma was higher in the anti-MDA5– group (*p* = 0.005).

**Table 1:** Comparison between the patients with idiopathic inflammatory myopathy (IIM) with or without positivity of anti-melanoma differentiation associated protein 5 (anti MDA5) antibody.

**Factors**	**MDA5 (+)**	**MDA5 (–)**	**P-value**
Age (y)	55.92 ± 7.68	56.88 ± 13.65	0.744
Sex (male/female)	8/18	20/23	0.197
Course of disease	2.00 (1.00, 3.25)	2.00 (1.00, 4.00)	0.694
Duration of diagnosis delay	1.25 (0.50, 2.00)	1.00 (1.00, 3.00)	0.537
Disease activity			
MYOACT score	13.00 (7.75, 16.00)	9.00 (7.00, 10.00)	0.003
Clinical manifestations or complications			
Dysphagia	5 (19.2%)	10 (23.3%)	0.694
Dysarthria	3 (11.5%)	4 (9.3%)	1.000
Respiratory muscle involvement	0 (0.0%)	0 (0.0%)	NA
Gastrointestinal hemorrhage	4 (15.4%)	1 (2.3%)	0.063
Fever	14 (53.8%)	14 (32.6%)	0.081
Hepatomegaly	1 (3.8%)	3 (7.0%)	1.000
Splenomegaly	10 (38.5%)	15 (34.9%)	0.764
Pulmonary bacterial infection	7 (26.9%)	5 (11.6%)	0.188
Pulmonary fungal infection	5 (19.2%)	4 (9.3%)	0.282
Tuberculosis infection	0 (0.0%)	1 (2.3%)	1.000
EBV infection	6 (23.1%)	8 (18.6%)	0.654
CMV infection	1 (3.8%)	2 (4.7%)	1.000
ILD	25 (96.2%)	36 (83.7%)	0.243
RP-ILD	11 (42.3%)	10 (23.3%)	0.096
Carcinoma	0 (0.0%)	11 (25.6%)	0.005
Lung function testing			
FVC% (%)	66.66 ± 19.22	71.66 ± 15.96	0.273
FEV1% (%)	66.71 ± 18.26	74.19 ± 19.23	0.132
FEV1/FVC	0.81 (0.75, 0.85)	0.81 (0.77, 0.85)	0.523
TLC (L)	3.37 ± 0.81	4.09 ± 1.09	0.007
DLCO% (%)	56.94 ± 17.86	64.14 ± 17.80	0.126
On-admission laboratory findings			
WBC (10^9^/L)	4.85 (3.20, 6.23)	8.10 (5.40, 10.90)	0.001
Neutrophils (10^9^/L)	3.40 (2.13, 5.45)	5.80 (3.80, 8.40)	0.002
Hemoglobin (g/L)	115.81 ± 17.33	120.72 ± 19.17	0.289
Platelets (10^9^/L)	165.00 ± 73.12	221.67 ± 81.89	0.005
Ferritin (ng/ml)	1112.60 (577.28, 3842.7)	511.40 (261.80, 1232.10)	0.012
CRP (mg/L)	4.00 (2.70, 7.43)	8.90 (3.20, 42.70)	0.010
ESR (mm/h)	20.50 (9.00, 45.75)	13.00 (6.00, 25.00)	0.185
ALT (U/L)	64.50 (31.00, 128.25)	84.00 (24.00, 139.00)	0.946
AST (U/L)	53.50 (37.00, 234.75)	57.00 (26.00, 141.00)	0.350
LDH (U/L)	307.00 (251.25, 382.00)	365.00 (264.00, 625.00)	0.144
CK (U/L)	81.00 (50.50, 297.75)	125.00 (59.00, 608.00)	0.244
PET/CT scan			
Bilateral lung SUVmax	0.72 (0.55, 0.87)	0.58 (0.52, 0.69)	0.029
Liver SUVmax	2.38 ± 0.52	2.20 ± 0.53	0.166
Spleen SUVmax	2.61 ± 0.67	2.24 ± 0.53	0.011
Bone marrow SUVmax	2.71 (2.40, 3.71)	2.61 (2.08, 2.99)	0.048
Cardiac SUVmax	1.76 (1.46, 2.39)	2.22 (1.34, 4.06)	0.400
Esophagus SUVmax	1.91 (1.42, 2.32)	1.64 (1.29, 2.27)	0.301
Stomach SUVmax	0.90 (0.78, 1.23)	0.95 (0.73, 1.21)	0.916
Small intestine SUVmax	1.62 ± 0.50	1.52 ± 0.40	0.342
Colon and rectum SUVmax	1.76 (1.32, 2.09)	1.46 (1.23, 2.08)	0.356
Bilateral cerebellum SUVmax	7.44 ± 2.20	6.59 ± 1.81	0.084
Bilateral trapezius SUVmax	0.96 (0.85, 1.17)	1.22 (0.93, 1.44)	0.053
Bilateral deltoid SUVmax	0.95 (0.76, 1.31)	1.01 (0.86, 1.36)	0.356
Bilateral biceps SUVmax	0.88 (0.71, 1.09)	1.21 (0.86, 1.50)	0.006
Bilateral iliopsoas SUVmax	1.24 (1.02, 1.65)	1.41 (1.07, 1.85)	0.353
Bilateral gluteus maximus SUVmax	1.05 (0.81, 1.27)	1.01 (0.84, 1.15)	0.762
Bilateral gluteus medius SUVmax	1.19 (0.95, 1.30)	1.22 (1.03, 1.62)	0.235
Bilateral quadriceps SUVmax	1.17 ± 0.36	1.27 ± 0.45	0.312
Therapeutic regimens			
Steroid monotherapy	4 (15.4%)	19 (44.2%)	0.014
Steroid+DMARDs	13 (50.0%)	15 (34.9%)	0.215
Steroid+IVIG	4 (15.4%)	3 (7.0%)	0.413
Steroid+DMARDs+IVIG	2 (7.7%)	5 (11.6%)	0.703
Steroid+JAK inhibitor	3 (11.5%)	1 (2.3%)	0.147
IIM subtypes			
DM	17 (65.4%)	28 (65.1%)	0.982
PM	0 (0.0%)	12 (27.9%)	0.002
ADM	9 (34.6%)	3 (7.0%)	0.007

*y, years; m, months; MYOACT, Myositis Disease Activity Assessment Visual Analog Scales; NA, not available; EBV, Epstein-Barr virus; CMV, cytomegalovirus; ILD, interstitial lung disease; RP-ILD, rapidly progressive interstitial lung disease; FVC%, percent-predicted forced vital capacity; FEV1%, percent-predicted forced expiratory volume in 1 s; FEV1/FVC, ratio of FEV1 over FVC; TLC, total lung capacity; DLCO%, percent-predicted diffusing capacity of the lung for carbon monoxide; WBC, white blood cell; CRP, C-reactive protein; ESR, erythrocyte sedimentation rate; ALT, alanine transaminase; AST, aspartate transaminase; LDH, lactate dehydrogenase; CK, creatine kinase; PET/CT, positron emission tomography/computed tomography; SUVmax, maximum standardized uptake value; DMARDs, disease-modifying antirheumatic drugs; IVIG, immunoglobulin; JAK, Janus kinase; IIM, idiopathic inflammatory myopathy; DM, dermatomyositis; PM, polymyositis; ADM, amyopathic dermatomyositis*.

The majority of the patients with anti-MDA5+ DM had ILD (96.2%) and a considerable proportion had RP-ILD (42.3%). About 83.7% patients had ILD in the anti-MDA5– group, with 23.3% patients had RP-ILD, owing to a considerable proportion of anti-synthetase syndrome (19/43). Furthermore, compared with the anti-MDA5– group, the patients in the anti-MDA5+ group had a higher MYOACT score (*p* = 0.003) and lower total lung capacity (*p* = 0.007).

**Figure 2:**
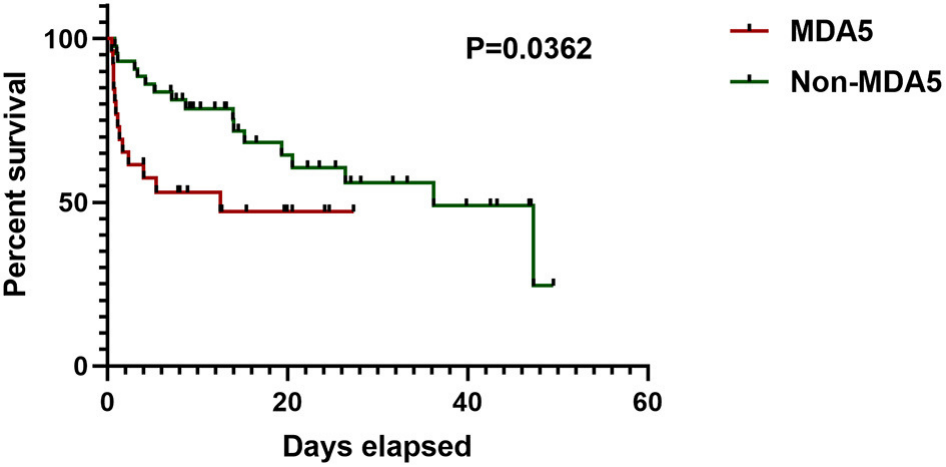
Survival of anti-MDA5+ group and anti-MDA5– group. MDA5, melanoma differentiation associated protein 5.

Compared with the anti-MDA5– group, the patients in the anti-MDA5+ group showed lower level of white blood cell (WBC, *p* = 0.001), neutrophil (*p* = 0.002), platelet (*p* = 0.005), C-reactive protein (CRP, *p* = 0.010), and higher level of ferritin (*p* = 0.012). There were no differences in serum alanine aminotransferase (ALT), serum aspartate aminotransferase (AST), nor differences in serum lactate dehydrogenase (LDH), or creatine kinase (CK) between the anti-MDA5+ and the anti-MDA5– group. Higher percentage of ADM (*p* = 0.007) was found in the anti-MDA5+ group. In the Kaplan–Meier analysis, the log-rank test demonstrated a significant difference (*p* = 0.0362) in survival between the anti-MDA5+ and the anti-MDA5– group (Figure 2).

Distribution of MSAs and MAAs in the anti-MDA5+/– groups are presented in Supplementary Table 1. MSAs were reported in all the patients. Anti-synthetase antibodies were positive in 19 cases in anti-MDA5– group: anti-PL7 antibody (*n* = 7), anti-PL12 antibody (*n* = 2), anti-EJ antibody (*n* = 2), anti-OJ antibody (*n* = 1), and anti-Jo 1 antibody (*n* = 7). Anti-TIF1γ antibodies were positive in six cases (*p* = 0.076) and anti-NXP2 antibodies were positive in eight cases (*p* = 0.021) in the anti-MDA5– group. MAAs were present in 22 cases in anti-MDA5– group: anti-Ro-52 antibody (*n* = 16), anti-PM-Scl75 antibody (*n* = 4), and anti-Ku antibody (*n* = 2).

### FDG PET/CT Scan Findings

**Figure 3:**
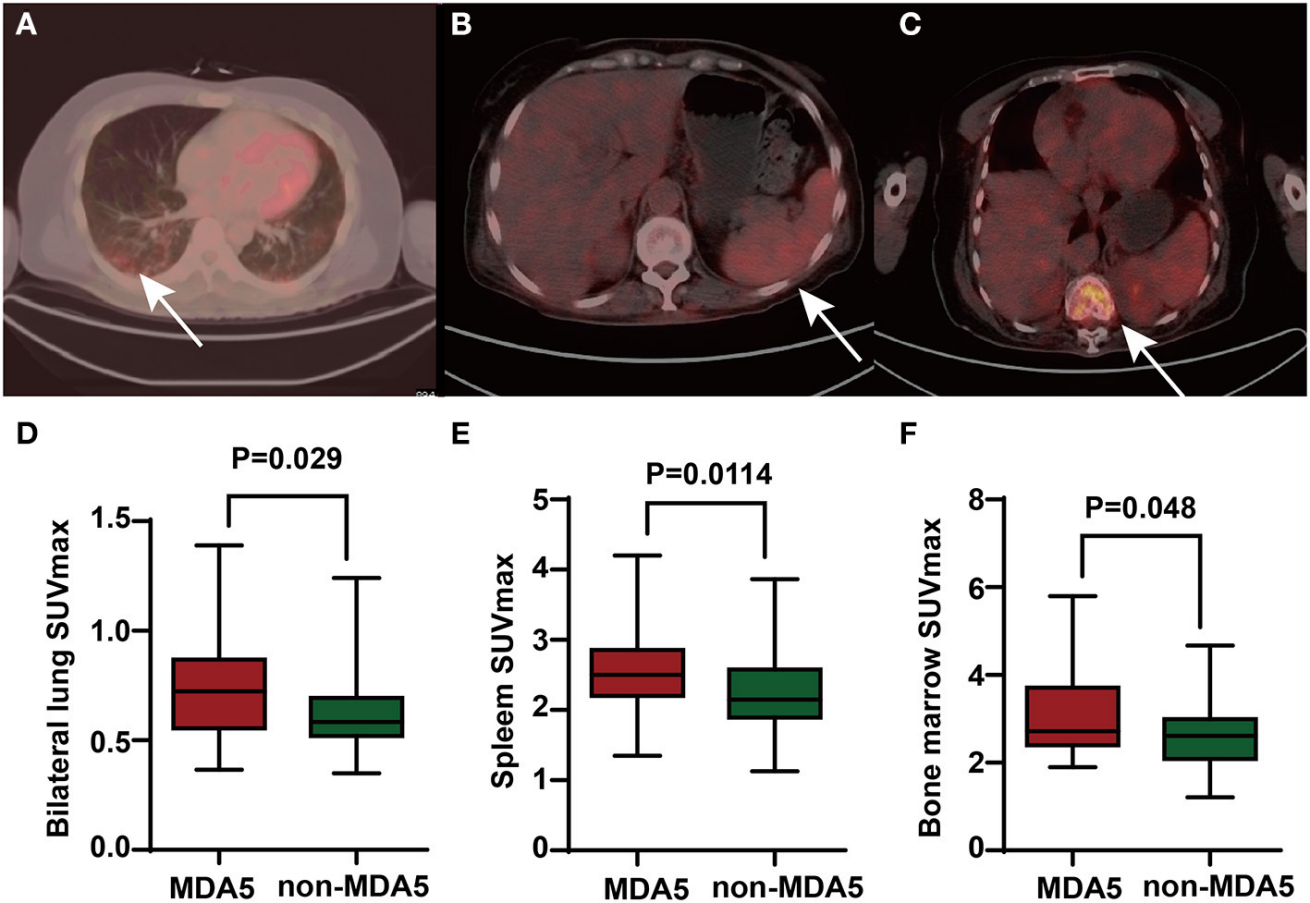
The PET/CT images of anti-MDA5+ DM patients and comparison of FDG uptake between anti-MDA5+ group and anti-MDA5– group. **(A)** Elevated FDG uptake of lungs (where the arrow pointed). **(B)** Elevated FDG uptake of spleen (where the arrow pointed). **(C)** Elevated FDG uptake of bone marrow (where the arrow pointed). **(D)** Comparison of bilateral lung SUVmax between the anti-MDA5+ group and anti-MDA5– group. **(E)** Comparison of spleen SUVmax between the anti-MDA5+ group and anti-MDA5– group. **(F)** Comparison of bone marrow SUVmax between the anti-MDA5+ group and anti-MDA5– group. SUVmax, maximum standardized uptake value; MDA5, melanoma differentiation associated protein 5; DM, dermatomyositis; FDG, ^18^F-fluorodeoxyglucose.

**Figure 4:**
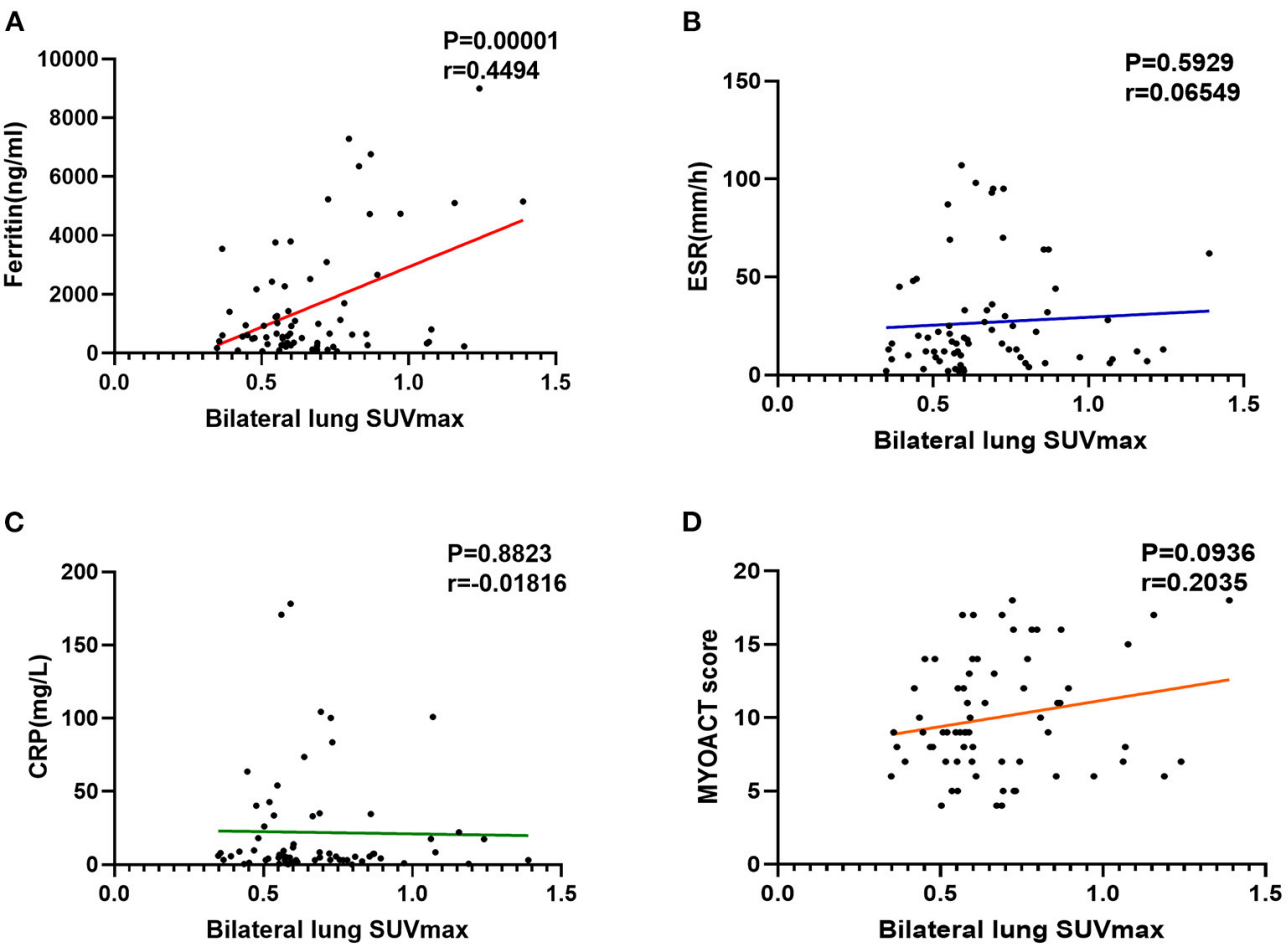
Evaluation of abnormal lung FDG uptake in the patients with anti-MDA5+ DM. **(A)** Correlation between ferritin and bilateral lung SUVmax. **(B)** Correlation between ESR and bilateral lung SUVmax. **(C)** Correlation between CRP and bilateral lung SUVmax. **(D)** Correlation between MYOACT score and bilateral lung SUVmax. FDG, ^18^F-fluorodeoxyglucose; MDA5, melanoma differentiation associated protein 5; DM, dermatomyositis; SUVmax, maximum standardized uptake value; ESR, Erythrocyte sedimentation rate; CRP, C-reactive protein; MYOACT, Myositis Disease Activity Assessment Visual Analog Scales.

By comparing the patients with anti-MDA5+ DM and anti-MDA5–, we found a higher bilateral lung SUVmax (*p* = 0.029) in anti-MDA5+ group (Figures 3A,D). Besides, a significant positive correlation between serum ferritin and bilateral lung SUVmax (*r* = 0.449, *p* < 0.001, Figure 4A) in the patients with anti-MDA5+ DM. However, no significant correlation was observed between the ESR, serum CRP levels, MYOACT score, and bilateral lung SUVmax (Figures 4B–D, respectively). These results suggest that FDG uptake (SUVmax) in the lungs by FDG-PET/CT imaging may be useful to evaluate the location and activity of ILD in the patients with anti-MDA5+ DM.

**Figure 5:**
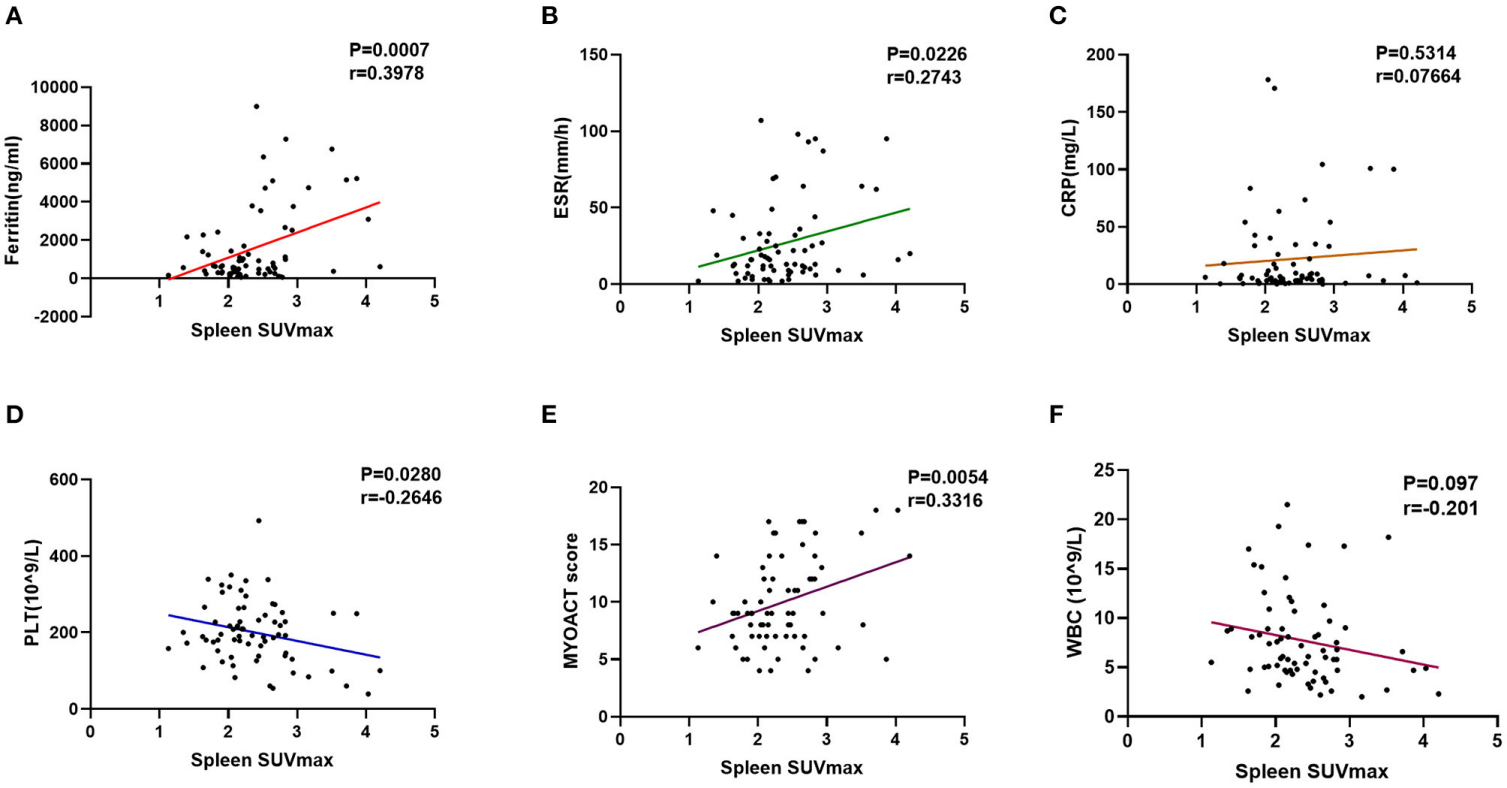
Evaluation of abnormal spleen FDG uptake in the patients with anti-MDA5+ DM. **(A)** Correlation between ferritin and spleen SUVmax. **(B)** Correlation between ESR and spleen SUVmax. **(C)** Correlation between CRP and spleen SUVmax. **(D)** Correlation between PLT and spleen SUVmax. **(E)** Correlation between MYOACT score and spleen SUVmax. **(F)** Correlation between WBC and spleen SUVmax. FDG, ^18^F-fluorodeoxyglucose; MDA5, melanoma differentiation associated protein 5; DM, dermatomyositis; SUVmax, maximum standardized uptake value; ESR, Erythrocyte sedimentation rate; CRP, C-reactive protein; PLT, platelet; MYOACT, Myositis Disease Activity Assessment Visual Analog Scales; WBC, white blood cell.

Interestingly, the patients with anti-MDA5+ DM were found to have significantly higher SUVmax of spleen (*p* = 0.011) and bone marrow (*p* = 0.048) than those in the patients with anti-MDA5– IIM (Figures 3B,C,E,F, respectively). Significant correlations between the spleen SUVmax and serum ferritin levels (*r* = 0.398, *p* < 0.001), ESR (*r* = 0.274, *p* = 0.023), platelet count (*r* = – 0.265, *p* = 0.028), and MYOACT score (*r* = 0.332, *p* = 0.005) were as well observed (Figures 5A,B,D,E, respectively). However, no significant correlation was identified between the spleen SUVmax and serum CRP/WBC levels (Figures 5C,F, respectively). In addition, we recognized a significantly positive correlation between the spleen SUVmax and bone marrow SUVmax (*r* = 0.564, *p* < 0.001, Supplementary Figure 1A), as well as bilateral lung SUVmax (*r* = 0.393, *p* < 0.001, Supplementary Figure 1B). However, there existed no significant correlation between the bone marrow SUVmax and bilateral lung SUVmax (Supplementary Figure 1C) levels. These results suggested that the spleen FDG uptakes (SUVmax) might be valuable in the assessment of disease activity in the patients with anti-MDA5+ DM.

The patients with anti-MDA5– IIM had higher SUVmax of bilateral biceps (*p* = 0.006). In addition, we could not detect significant differences in SUVmax of the other organs: liver, heart, digestive system, cerebellum, and other proximal limb muscles between the patients with anti-MDA5+ and anti-MDA5– IIM (Table 1).

To explore the predictive value of PET/CT scans for the unfavorable short-term outcome (death within 3 months), we further analyzed the clinical data of 69 patients with IIM. The spleen SUVmax was found to be significantly correlated with the unfavorable short-term outcome (*p* = 0.001) (Supplementary Table 2). A ROC curve analysis revealed an area under the curve of 0.732 and a cutoff value of 2.319 (Supplementary Figure 2A). In addition, the spleen SUVmax was found to be significantly correlated with RP-ILD (*p* = 0.031) (Supplementary Table 3). A ROC curve analysis revealed an area under the curve of 0.684 and a cut-off value of 2.222 (Supplementary Figure 2B).

## Discussion

To the best of our knowledge, this is the first retrospective cohort study reported the PET/CT characteristics of the patients with anti-MDA5+ DM. A previous case report mentioned that ^18^F-FDG PET/CT was capable of detecting bilateral pulmonary inflammatory 1 month prior to the development of RP-ILD in a patient with an anti-MDA5+ DM (23). However, the PET/CT characteristics remain unclear in the patients with anti-MDA5+ DM.

F-Fluorodeoxyglucose PET/CT had shown multiple diagnostic values for the patients IIM, the main interest of which focused on cancer screening and measures of myositis activity (24). In the patients with IIM, a good correlation was found between the proximal muscle SUVmax and the serum creatine kinase, muscle strength, muscle biopsy findings in several studies (13, 14, 25). ^18^F-FDG PET/CT can help to verify the muscle inflammation activity in IIM. However, lower sensitivity for the myositis diagnosis was reported for ^18^F-FDG PET/CT imaging compared with the conventional examinations, such as muscle electrophysiological examinations and MRI (24).

In this retrospective study, the patients with anti-MDA5+ DM were found to suffer more prominent pulmonary inflammation in 18F-FDG PET/CT than those with anti-MDA5–. The evaluating role of PET/CT in pulmonary focal inflammation had been demonstrated in the preceding studies. Uehara et al. reported that the ^18^F-FDG FDG SUVmax changed after treatment and was consistent with the activity of ILD (26). The patients with progressive disease in Granulomatous-Lymphocytic ILD had significantly higher SUVmean on ^18^F-FDG-PET/CT which suggested that this modality may be valuable for identifying the patients with active pulmonary inflammation and progressive disease (27). ^18^F-FDG-PET/CT was found to be useful to evaluate the pulmonary inflammation in other connective tissue disease associated ILD, such as systemic sclerosis (28, 29).

Notably, the patients with anti-MDA5+ DM had a higher FDG uptake of the spleen which was significantly associated with myositis disease activity and pulmonary inflammation. Likewise, these findings might suggest that the patients with anti-MDA5+ DM had distinctive characteristics, and potentially represents unique pathophysiological mechanisms. Given that serum ferritin has been used as a marker of macrophage activation (30), we presumed that spleen SUVmax of the patients with anti-MDA5+ may be related to macrophage activation. In this study, we observed that the patients with anti-MDA5+ showed some similarities with macrophage activation syndrome (MAS), such as leukocytopenia, thrombocytopenia, elevated serum ferritin, and splenomegaly, but were not fully compliant with the diagnostic criteria of MAS. Zuo et al. (10) reported that the infiltration of CD163-positive macrophages into alveolar spaces was significantly higher in RP-ILD group of the patients with DM, which as well-indicated that macrophage activation might be involved in the pathogenesis of RP-ILD in DM.

Although the pathophysiology of anti-MDA5+ DM is still not fully understood, a widely accepted guess is an abnormal inflammatory response which led to multi-system involvement (lungs in particular). On the one hand, the alveolar cell damage is a consequence of systemic hyper-inflammation, on the other hand, if the integrity of the epithelial–endothelial lining is damaged, the alveolar macrophages will produce proinflammatory cytokines and chemokines, thus resulting in a cytokine storm. More recently, the association between the choline phospholipid metabolism and macrophage immune responsiveness has been identified. ^18^F-Fluorocholine was reported to image and quantify the macrophage activity in pulmonary interstitial infiltrates of coronavirus disease 2019 (COVID-19) pneumonia (31, 32), which might inspire similar investigation in the patients with anti-MDA5+ DM. In addition, Fibroblast Activation Protein specific PET/CT imaging was performed as a promising new imaging modality for fibrotic ILD and lung cancer (33). Its potential clinical value for monitoring and therapy evaluation of CTD-ILD should be investigated in the future studies.

The present study has several limitations, such as retrospective nature and the small sample size. The HRCT scans were evaluated qualitatively, instead of quantitative analyses. ^18^F-FDG PET/CT was identified as an important diagnostic tool when evaluating active inflammation and disease progression in the patients with anti-MDA5+ DM. A prospective cohort study with larger sample will be essential to confirm our findings and fill in the gaps.

## Conclusion

^18^F-FDG PET/CT was found valuable in quantifying the pulmonary focal inflammation and potentially unveiling the distinctive characteristics and pathophysiological mechanisms in the patients with anti-MDA5+ DM.

## Data Availability Statement

The raw data supporting the conclusions of this article will be made available by the authors, without undue reservation.

## Ethics Statement

The studies involving human participants were reviewed and approved by Institutional Review Board of the First Affiliated Hospital, Zhejiang University School of Medicine. The patients/participants provided their written informed consent to participate in this study. Written informed consent was obtained from the individual(s) for the publication of any potentially identifiable images or data included in this article.

## Author Contributions

JLin: conceptualization and methodology. HC: data curation and writing-original draft preparation. DX and JLia: verification of IIM diagnosis. YL: evaluation of FDG uptake in multiple organs. HC and YY: identification of ILD and RP-ILD. YS: writing and reviewing. YH: investigation and validation. All authors contributed to the article and approved the submitted version.

## Funding

This research was supported by the National Natural Science Foundation of China (Grant No. 81701602).

## Conflict of Interest

The authors declare that the research was conducted in the absence of any commercial or financial relationships that could be construed as a potential conflict of interest.

## Publisher's Note

All claims expressed in this article are solely those of the authors and do not necessarily represent those of their affiliated organizations, or those of the publisher, the editors and the reviewers. Any product that may be evaluated in this article, or claim that may be made by its manufacturer, is not guaranteed or endorsed by the publisher.

## References

[1] Dalakas MC. Inflammatory muscle diseases. N Engl J Med. (2015) 372:1734–47. 10.1056/NEJMra1402225 25923553

[2] Satoh M Tanaka S Ceribelli A Calise SJ Chan EK. A comprehensive overview on myositis-specific antibodies: new and old biomarkers in idiopathic inflammatory myopathy. Clin Rev Allergy Immunol. (2017) 52:1–19. 10.1007/s12016-015-8510-y 26424665 PMC5828023

[3] Fujimoto M Watanabe R Ishitsuka Y Okiyama N. Recent advances in dermatomyositis-specific autoantibodies. Curr Opin Rheumatol. (2016) 28:636–44. 10.1097/BOR.0000000000000329 27533321

[4] Kurtzman DJB Vleugels RA. Anti-melanoma differentiation-associated gene 5 (MDA5) dermatomyositis: a concise review with an emphasis on distinctive clinical features. J Am Acad Dermatol. (2018) 78:776–85. 10.1016/j.jaad.2017.12.010 29229575

[5] Wu W Guo L Fu Y Wang K Zhang D Xu W. Interstitial lung disease in Anti-MDA5 positive dermatomyositis. Clin Rev Allergy Immunol. (2021) 60:293–304. 10.1007/s12016-020-08822-5 33405101

[6] Allenbach Y Uzunhan Y Toquet S Leroux G Gallay L Marquet A. Different phenotypes in dermatomyositis associated with anti-MDA5 antibody: study of 121 cases. Neurology. (2020) 95:e70–8. 10.1212/WNL.0000000000009727 32487712 PMC7371381

[7] Li Y Li Y Wu J Miao M Gao X Cai W. Predictors of poor outcome of Anti-MDA5-associated rapidly progressive interstitial lung disease in a Chinese Cohort with dermatomyositis. J Immunol Res. (2020) 2020:2024869. 10.1155/2020/2024869 33299896 PMC7710415

[8] Motegi SI Sekiguchi A Toki S Kishi C Endo Y Yasuda M. Clinical features and poor prognostic factors of anti-melanoma differentiation-associated gene 5 antibody-positive dermatomyositis with rapid progressive interstitial lung disease. Eur J Dermatol. (2019) 29:511–7. 10.1684/ejd.2019.3634 31617496

[9] Ye Y Fu Q Wang R Guo Q Bao C. Serum KL-6 level is a prognostic marker in patients with anti-MDA5 antibody-positive dermatomyositis associated with interstitial lung disease. J Clin Lab Anal. (2019) 33:e22978. 10.1002/jcla.22978 31301087 PMC6805307

[10] Zuo Y Ye L Liu M Li S Liu W Chen F. Clinical significance of radiological patterns of HRCT and their association with macrophage activation in dermatomyositis. Rheumatology. (2020) 59:2829–37. 10.1093/rheumatology/keaa034 32065646

[11] Selva-O'Callaghan A Gil-Vila A Simo-Perdigo M Trallero-Araguas E Alvarado-Cardenas M Pinal-Fernandez I. PET scan: nuclear medicine imaging in myositis. Curr Rheumatol Rep. (2019) 21:64. 10.1007/s11926-019-0864-3 31754890 PMC11611048

[12] Matuszak J Blondet C Hubele F Gottenberg JE Sibilia J Bund C. Muscle fluorodeoxyglucose uptake assessed by positron emission tomography-computed tomography as a biomarker of inflammatory myopathies disease activity. Rheumatology. (2019) 58:1459–64. 10.1093/rheumatology/kez447 30851092

[13] Tanaka S Ikeda K Uchiyama K Iwamoto T Sanayama Y Okubo A. [18F]FDG uptake in proximal muscles assessed by PET/CT reflects both global and local muscular inflammation and provides useful information in the management of patients with polymyositis/dermatomyositis. Rheumatology. (2013) 52:1271–8. 10.1093/rheumatology/ket112 23479721

[14] Sun L Dong Y Zhang N Lv X Chen Q Wei W. [(18)F]Fluorodeoxyglucose positron emission tomography/computed tomography for diagnosing polymyositis/dermatomyositis. Exp Ther Med. (2018) 15:5023–8. 10.3892/etm.2018.6066 29805526 PMC5958694

[15] Li Y Zhou Y Wang Q. Multiple values of (18)F-FDG PET/CT in idiopathic inflammatory myopathy. Clin Rheumatol. (2017) 36:2297–305. 10.1007/s10067-017-3794-3 28831580

[16] Liang J Cao H Liu Y Ye B Sun Y Ke Y. The lungs were on fire: a pilot study of (18)F-FDG PET/CT in idiopathic-inflammatory-myopathy-related interstitial lung disease. Arthritis Res Ther. (2021) 23:198. 10.1186/s13075-021-02578-9 34301306 PMC8298695

[17] Lundberg IE Tjarnlund A Bottai M Werth VP Pilkington C Visser M. 2017 European League Against Rheumatism/American College of Rheumatology classification criteria for adult and juvenile idiopathic inflammatory myopathies and their major subgroups. Ann Rheum Dis. (2017) 76:1955–64. 10.1136/annrheumdis-2017-211468 29079590 PMC5736307

[18] Rider LG Werth VP Huber AM Alexanderson H Rao AP Ruperto N. Measures of adult and juvenile dermatomyositis, polymyositis, and inclusion body myositis: Physician and Patient/Parent Global Activity, Manual Muscle Testing (MMT), Health Assessment Questionnaire (HAQ)/Childhood Health Assessment Questionnaire (C-HAQ), Childhood Myositis Assessment Scale (CMAS), Myositis Disease Activity Assessment Tool (MDAAT), Disease Activity Score (DAS), Short Form 36 (SF-36), Child Health Questionnaire (CHQ), physician global damage, Myositis Damage Index (MDI), Quantitative Muscle Testing (QMT), Myositis Functional Index-2 (FI-2), Myositis Activities Profile (MAP), Inclusion Body Myositis Functional Rating Scale (IBMFRS), Cutaneous Dermatomyositis Disease Area and Severity Index (CDASI), Cutaneous Assessment Tool (CAT), Dermatomyositis Skin Severity Index (DSSI), Skindex, and Dermatology Life Quality Index (DLQI). Arthritis Care Res. (2011) 63(Suppl. 11):S118–57. 10.1002/acr.20532 22588740 PMC3748930

[19] American Thoracic S, European Respiratory S. American Thoracic Society/European Respiratory Society International Multidisciplinary Consensus Classification of the Idiopathic Interstitial Pneumonias. This joint statement of the American Thoracic Society (ATS), and the European Respiratory Society (ERS) was adopted by the ATS board of directors, June 2001 and by the ERS Executive Committee, June 2001. Am J Respir Crit Care Med. (2002) 165:277–304. 10.1164/ajrccm.165.2.ats01 11790668

[20] Horiike Y Suzuki Y Fujisawa T Yasui H Karayama M Hozumi H. Successful classification of macrophage-mannose receptor CD206 in severity of anti-MDA5 antibody positive dermatomyositis associated ILD. Rheumatology. (2019) 58:2143–52. 10.1093/rheumatology/kez185 31143953

[21] Abe Y Matsushita M Tada K Yamaji K Takasaki Y Tamura N. Clinical characteristics and change in the antibody titres of patients with anti-MDA5 antibody-positive inflammatory myositis. Rheumatology. (2017) 56:1492–7. 10.1093/rheumatology/kex188 28499006

[22] Boellaard R Delgado-Bolton R Oyen WJ Giammarile F Tatsch K Eschner W. FDG PET/CT: EANM procedure guidelines for tumour imaging: version 2.0. Eur J Nucl Med Mol Imaging. (2015) 42:328–54. 10.1007/s00259-014-2961-x 25452219 PMC4315529

[23] Morita Y Kuwagata S Kato N Tsujimura Y Mizutani H Suehiro M. 18F-FDG PET/CT useful for the early detection of rapidly progressive fatal interstitial lung disease in dermatomyositis. Intern Med. (2012) 51:1613–8. 10.2169/internalmedicine.51.6813 22728501

[24] Owada T Maezawa R Kurasawa K Okada H Arai S Fukuda T. Detection of inflammatory lesions by f-18 fluorodeoxyglucose positron emission tomography in patients with polymyositis and dermatomyositis. J Rheumatol. (2012) 39:1659–65. 10.3899/jrheum.111597 22753657

[25] Tateyama M Fujihara K Misu T Arai A Kaneta T Aoki M. Clinical values of FDG PET in polymyositis and dermatomyositis syndromes: imaging of skeletal muscle inflammation. BMJ Open. (2015) 5:e006763. 10.1136/bmjopen-2014-006763 25582454 PMC4298089

[26] Uehara T Takeno M Hama M Yoshimi R Suda A Ihata A. Deep-inspiration breath-hold 18F-FDG-PET/CT is useful for assessment of connective tissue disease associated interstitial pneumonia. Mod Rheumatol. (2016) 26:121–7. 10.3109/14397595.2015.1054099 25995034

[27] Fraz MSA Moe N Revheim ME Stavrinou ML Durheim MT Nordoy I. Granulomatous-lymphocytic interstitial lung disease in common variable immunodeficiency-features of CT and (18)F-FDG positron emission tomography/CT in clinically progressive disease. Front Immunol. (2020) 11:617985. 10.3389/fimmu.2020.617985 33584710 PMC7874137

[28] Bellando-Randone S Tartarelli L Cavigli E Tofani L Bruni C Lepri G. 18F-fluorodeoxyglucose positron-emission tomography/CT and lung involvement in systemic sclerosis. Ann Rheum Dis. (2019) 78:577–8. 10.1136/annrheumdis-2018-213376 30337426

[29] Ledoult E Morelle M Soussan M Mekinian A Behal H Sobanski V. (18)F-FDG positron emission tomography scanning in systemic sclerosis-associated interstitial lung disease: a pilot study. Arthritis Res Ther. (2021) 23:76. 10.1186/s13075-021-02460-8 33673861 PMC7936499

[30] Truman-Rosentsvit M Berenbaum D Spektor L Cohen LA Belizowsky-Moshe S Lifshitz L. Ferritin is secreted via 2 distinct nonclassical vesicular pathways. Blood. (2018) 131:342–52. 10.1182/blood-2017-02-768580 29074498 PMC5774206

[31] Savelli G Bonacina M Rizzo A Zaniboni A. Activated macrophages are the main inflammatory cell in COVID-19 interstitial pneumonia infiltrates. Is it possible to show their metabolic activity and thus the grade of inflammatory burden with (18)F-Fluorocholine PET/CT? Med Hypotheses. (2020) 144:109885. 10.1016/j.mehy.2020.109885 32540605 PMC7252431

[32] Turpin L Pouliot Q Zhang J Glikman M Gomez F Talbot JN. (18)F-Fluorocholine uptake matching CT lesions in the lungs of a patient clinically cured from COVID-19 syndrome. Eur J Nucl Med Mol Imaging. (2020) 47:2706–8. 10.1007/s00259-020-04919-3 32561970 PMC7304916

[33] Rohrich M Leitz D Glatting FM Wefers AK Weinheimer O Flechsig P. Fibroblast activation protein specific PET/CT imaging in fibrotic interstitial lung diseases and lung cancer: a translational exploratory study. J Nucl Med. (2021). 10.2967/jnumed.121.261925. [Epub ahead of print]. 34272325 PMC8717194

