# Supplementary material for: Radiological Characteristics of Patients With Anti-MDA5–Antibody-Positive Dermatomyositis in 18F-FDG PET/CT: A Pilot Study
Source: Front Med (Lausanne). 2021 Nov 22;8:779272. doi: 10.3389/fmed.2021.779272 (PMC8645547; doi:10.3389/fmed.2021.779272)
Supplement: Supplementary file 1 [file Data_Sheet_1.pdf]

Supplementary table 1 Distribution of MSAs and MAAs in anti-MDA5-positive/negative groups.

MSAs: Myositis specific antibodies; MAAs: Myositis associated antibodies; N: Number.

MSAs or MAAs	Anti-MDA5-positive (26)	Anti-MDA5-negative (43)	P value
Anti-PL-7	0(0.0%)	7(16.3%)	0.040
Anti-PL-12	0(0.0%)	2(4.7%)	0.523
Anti-EJ	0(0.0%)	2(4.7%)	0.523
Anti-OJ	0(0.0%)	1(2.3%)	1.000
Anti-Jo-1	0(0.0%)	7(16.3%)	0.040
Anti-TIF1γ	0(0.0%)	6(14.0%)	0.076
Anti-Mi-2α	0(0.0%)	2(4.7%)	0.523
Anti-Mi-2β	0(0.0%)	4(9.3%)	0.289
Anti-SAE1	0(0.0%)	6(14.0%)	0.076
Anti-NXP2	0(0.0%)	8(18.6%)	0.021
Anti-SRP	0(0.0%)	3(7.0%)	0.285
Anti-Ku	0(0.0%)	2(4.7%)	0.523
Anti-PM-Scl75	0(0.0%)	4(9.3%)	0.289
Anti-PM-Scl100	0(0.0%)	0(0.0%)	NA
Anti-Ro-52	17(65.4%)	16(37.2%)	0.028

Supplementary table 2 Correlation between focal FDG uptake and unfavorable outcome

FDG: ^{18}F-Fluorodeoxyglucose; SUVmax: maximum standardized uptake value.

Factors	≤3 months (14)	>3 months (55)	P value
Bilateral lung SUVmax	0.76±0.30	0.65±0.19	0.112
Liver SUVmax	2.28±0.35	2.27±0.57	0.953
Spleen SUVmax	2.84±0.72	2.26±0.52	0.001
Bone marrow SUVmax	2.69(2.29,3.30)	2.67(2.22,3.35)	0.864
Cardiac SUVmax	1.76(1.30,3.08)	2.06(1.40,3.63)	0.806
Esophagus SUVmax	1,78(1.41,2.58)	1.71(1.30,2.27)	0.483
Stomach SUVmax	0.96(0.60,1.31)	0.93(0.82,1.23)	0.893
Small intestine SUVmax	1.70±0.32	1.52±0.46	0.162
Colon and rectum SUVmax	1.54(1.36,2.03)	1.55(1.25,2.08)	0.743
Bilateral cerebellum SUVmax	6.80±1.85	6.94±2.04	0.819
Bilateral trapezius SUVmax	1.14(0.87,1.44)	1.11(0.87,1.32)	0.571
Bilateral deltoid SUVmax	0.90(0.78,1.40)	1.01(0.83,1.35)	0.576
Bilateral biceps SUVmax	0.89(0.67,1.18)	1.08(0.84,1.46)	0.102
Bilateral ilioposas SUVmax	1.20(1.08,1.65)	1.41(0.97,1.85)	0.465
Bilateral gluteus maximus SUVmax	0.98(0.83,1.37)	1.03(0.78,1.18)	0.693
Bilateral gluteus medius SUVmax	1.23(1.04,1.40)	1.20(0.99,1.51)	0.881
Bilateral quadriceps SUVmax	1.22±0.35	1.23±0.43	0.933

Supplementary table 3 Correlation between focal FDG uptake and RP-ILD (within 61 IIM-ILD patients)

FDG: ^{18}F-Fluorodeoxyglucose; RP-ILD: rapidly progressive interstitial lung disease; SUVmax: maximum standardized uptake value.

Factors	RP-ILD (21)	Non-RP-ILD (40)	P value
**Bilateral lung SUVmax**	**0.77±0.21**	**0.66±0.22**	**0.059**
**Liver SUVmax**	**2.46±0.43**	**2.26±0.55**	**0.158**
**Spleen SUVmax**	**2.64±0.62**	**2.28±0.60**	**0.031**
**Bone marrow SUVmax**	**2.70(2.38,3.16)**	**2.61(2.17,3.29)**	**0.524**
**Cardiac SUVmax**	**1.73(1.32,2.91)**	**2.20(1.59,3.92)**	**0.448**
**Esophagus SUVmax**	**1.88(1.52,2.66)**	**1.69(1.32,2.26)**	**0.149**
**Stomach SUVmax**	**0.89(0.62,1.43)**	**0.95(0.85,1.12)**	**0.796**
**Small intestine SUVmax**	**1.59±0.49**	**1.56±0.44**	**0.807**
**Colon and rectum SUVmax**	**1.65(1.37,2.47)**	**1.59(1.25,2.06)**	**0.362**
**Bilateral cerebellum SUVmax**	**6.91±1.52**	**7.08±2.20**	**0.752**
**Bilateral trapezius SUVmax**	**1.13(0.88,1.28)**	**1.03(0.83,1.28)**	**0.509**
**Bilateral deltoid SUVmax**	**0.88(0.75,1.30)**	**1.00(0.84,1.34)**	**0.295**
**Bilateral biceps SUVmax**	**0.91(0.77,1.24)**	**0.97(0.82,1.34)**	**0.509**
**Bilateral ilioposas SUVmax**	**1.14(1.01,1.76)**	**1.29(0.99,1.77)**	**0.855**
**Bilateral gluteus maximus SUVmax**	**1.09(0.90,1.39)**	**1.02(0.77,1.14)**	**0.065**
**Bilateral gluteus medius SUVmax**	**1.18(0.98,1.36)**	**1.20(1.01,1.46)**	**0.779**
**Bilateral quadriceps SUVmax**	**1.18±0.36**	**1.22±0.39**	**0.679**

Supplementary figure 1 Correlation of spleen SUVmax, bilateral lung SUVmax and bone marrow SUVmax.

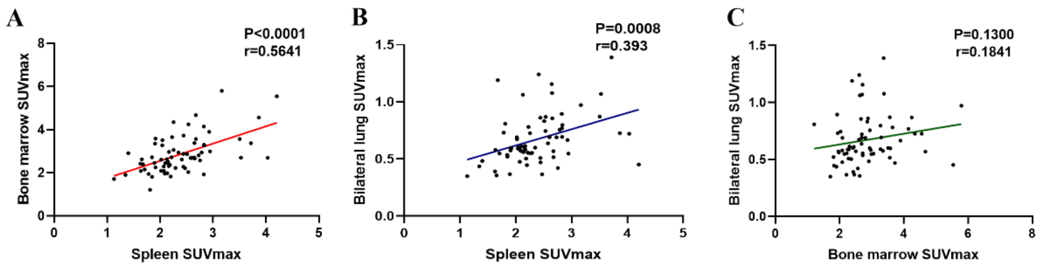

SUVmax: maximum standardized uptake value

Supplementary figure 2 The predictive value of PET/CT for unfavorable outcome and RP-ILD

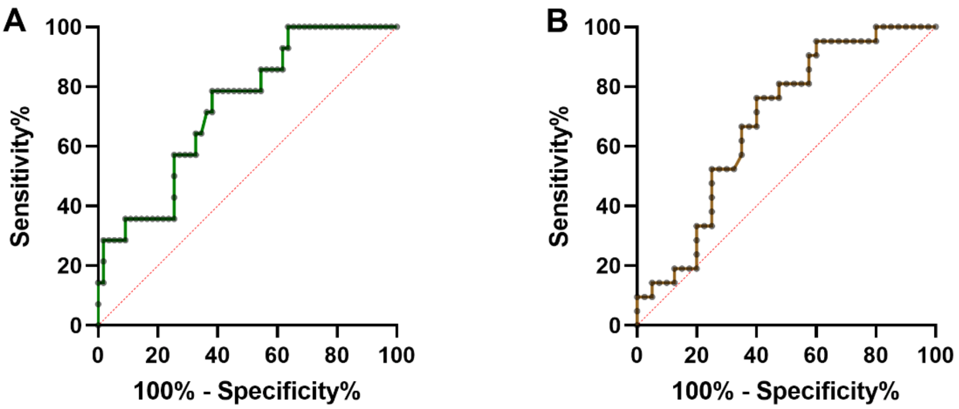

A. ROC curve of spleen SUVmax predicting unfavorable outcome (death within three months)

ROC=0.732; Cut off value= 2.319; Sensitivity= 78.6%; Specificity= 61.8%.

B. ROC curve of spleen SUVmax predicting RP-ILD

ROC=0.684; Cut off value= 2.222; Sensitivity= 76.2%; Specificity= 60.0%;

RP-ILD: rapidly progressive interstitial lung disease; SUVmax: maximum standardized uptake value; ROC: Receiver operating characteristic; AUC: Area under the curve.
